# Supplementary material for: Altered transcriptional and chromatin responses to rhinovirus in bronchial epithelial cells from adults with asthma
Source: Commun Biol. 2020 Nov 13;3:678. doi: 10.1038/s42003-020-01411-4 (PMC7666152; doi:10.1038/s42003-020-01411-4)
Supplement: Supplementary file 1 — Supplementary Information [file 42003_2020_1411_MOESM1_ESM.pdf]

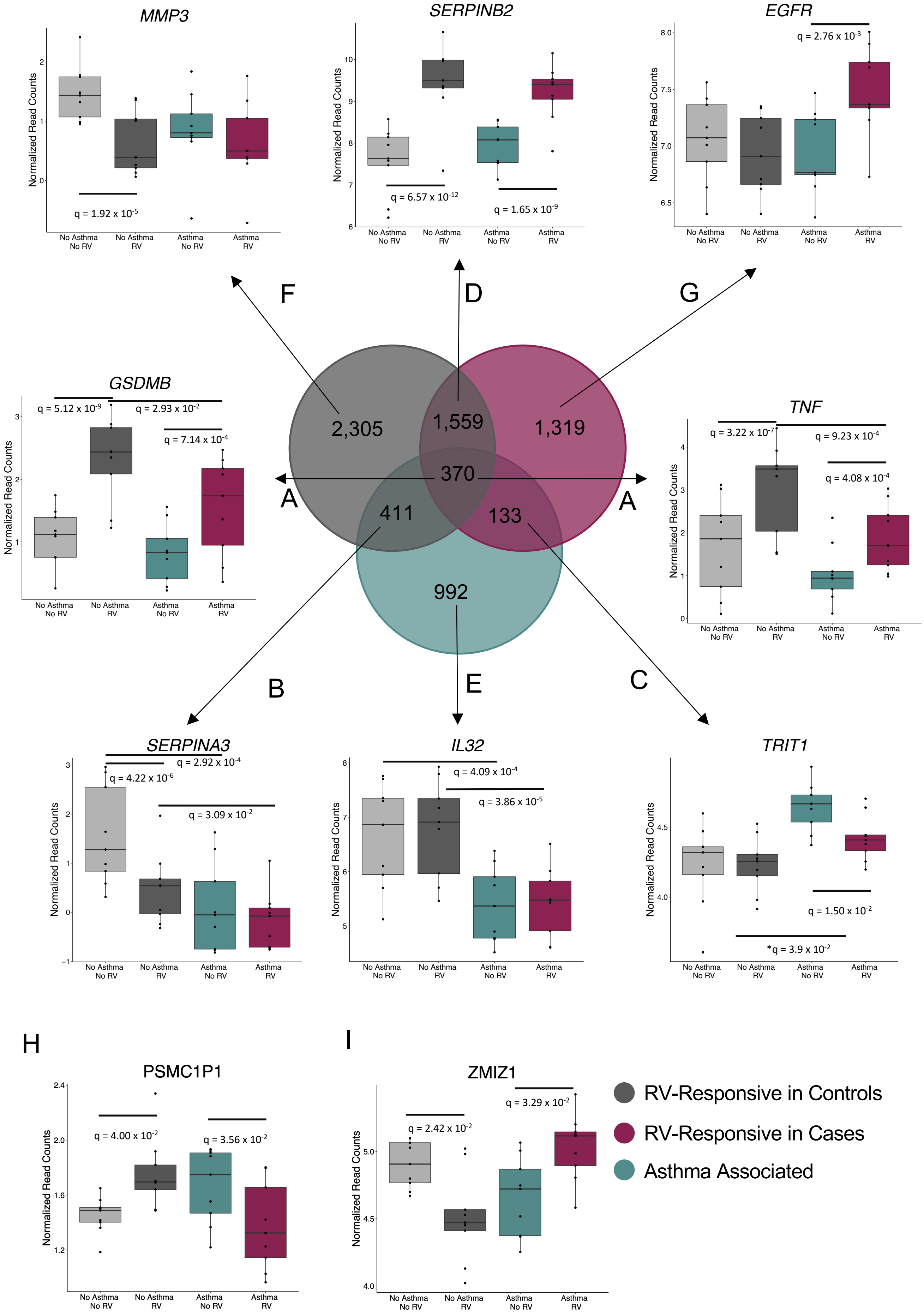

Supplementary Figure 1. Examples of different patterns of RV response in cases and controls.

Supplementary Table 1: Genes with Opposite Direction of Response to RV in Cases and Controls

	RV Response in Controls			RV Response in Cases		
**Gene**	**log_2FC**	**p-value**	**adj p-val**	**log_2FC**	**p-value**	**adj p-val**
ANKRD18B	-0.49	1.5E-03	1.1E-02	0.41	8.2E-03	2.9E-02
C1orf131	-0.25	6.9E-03	3.4E-02	0.26	1.2E-02	3.9E-02
CWC27	-0.26	1.0E-02	4.4E-02	0.25	8.5E-03	3.0E-02
FAM72B	-0.26	8.6E-03	3.9E-02	0.33	9.5E-03	3.2E-02
LEO1	-0.24	1.2E-02	4.9E-02	0.32	8.9E-03	3.1E-02
MPHOSPH10	-0.30	8.2E-04	7.2E-03	0.25	8.4E-03	3.0E-02
PSMC1P1	-0.29	7.5E-03	3.6E-02	0.30	1.3E-02	4.0E-02
PTP4A2	-0.16	9.5E-03	4.2E-02	0.16	1.7E-02	4.9E-02
RPIA	-0.30	9.8E-03	4.3E-02	0.38	7.3E-03	2.6E-02
SET	-0.27	2.0E-03	1.4E-02	0.24	8.2E-03	2.9E-02
SREK1	-0.19	1.2E-02	5.0E-02	0.32	2.3E-04	1.8E-03
ZNF326	-0.32	3.9E-04	4.1E-03	0.25	1.0E-02	3.4E-02
ZMIZ1	0.37	6.7E-03	3.3E-02	-0.39	6.5E-03	2.4E-02

Supplementary Table 2. RV-Responsive Areas of Open Chromatin and Genes Expression in Cases, Pairs Defined by Hi-C

Gene Name	Ensembl Gene ID	Gene Start	Gene End	Area of Open Chromatin	Spearman's Correlation (p-value)	Spearman's Correlation (FDR)	r.squared
OAS1	ENSG00000089127.12_2	113344582	113369990	chr12 113415907 - 113416948	1.70E-06	7.39E-04	0.461
OAS3	ENSG00000111331.12_1	113376157	113411054	chr12 113415907 - 113416948	3.56E-06	7.73E-04	0.493
LIFR	ENSG00000113594.9_1	38475065	38608456	chr5 39073392 - 39075336	7.90E-04	2.64E-02	0.344
NT5E	ENSG00000135318.11_2	86159809	86205500	chr6 86116163 - 86116824	1.68E-03	4.33E-02	0.274
NTPCR	ENSG00000135778.11_1	233086351	233119628	chr1 232765116 - 232766707	2.03E-03	4.90E-02	0.163
ASNSD1	ENSG00000138381.9_2	190526111	190535557	chr2 190305458 - 190306720	1.09E-03	3.38E-02	0.298
HERC6	ENSG00000138642.14_2	89299891	89364263	chr4 89377812 - 89378998	5.98E-06	8.67E-04	0.454
ZCCHC2	ENSG00000141664.9_2	60190240	60254942	chr18 60382070 - 60384909	1.82E-05	1.97E-03	0.292
RNF144A	ENSG00000151692.14_1	7057523	7208417	chr2 7017491 - 7018434	1.62E-04	7.84E-03	0.405
RABGAP1L	ENSG00000152061.23_2	174128548	174964445	chr1 173445760 - 173447549	1.58E-04	7.84E-03	0.327
JMY	ENSG00000152409.8_1	78532012	78623038	chr5 78809164 - 78810826	4.40E-04	1.59E-02	0.439
PPM1K	ENSG00000163644.14_2	89178772	89205921	chr4 89377812 - 89378998	6.66E-05	5.80E-03	0.444
NAA16	ENSG00000172766.18_1	41885341	41951166	chr13 41634161 - 41636298	1.48E-04	7.84E-03	0.368
FAM118B	ENSG00000197798.8_2	126081309	126132881	chr11 125931620 - 125933474	3.60E-04	1.42E-02	0.276

Supplementary Table 3: Correlated Chromatin-Gene Pairs in GWAS loci

**Gene-Chromatin Pair**		**adj. p-value**	**GWAS Locus**
PPP1R18	chr6 30614623 - 30615707	1.47E-02	chr6 30075103 - 33536633
TUBB	chr6 30614623 - 30615707	4.49E-02	chr6 30075103 - 33536633
PSMB8	chr6 32820984 - 32822286	3.85E-02	chr6 30075103 - 33536633
PRKCQ	chr10 6130645 - 6132004	2.55E-02	chr10 6618822 - 6631223
OVOL1	chr11 65600770 - 65601599	2.56E-02	chr11 65547333 - 65559266
STARD3	chr17 37910041 - 37911078	4.08E-02	chr17 37281157 - 38876841
FAM117A	chr17 47438647 - 47440059	4.50E-02	chr17 47320418 - 47461433
KAT7	chr17 47438647 - 47440059	3.57E-02	chr17 47320418 - 47461433

Supplementary Table 4

Reference Guide for Columns in Supplementary Data 5 .csv file.

Supplementary Data 5 Column Names	Descriptions
Motif.Name	Motif Identifier with transcription factor
Num.of.Target.Sequences.with.Motif	The number of regions containing the motif
Percent.of.Target.Sequences.with.Motif	Percent of all regions that contain the motif
Num.of.Background.Sequences.with.Motif	The number of regions in the background--here all ATAC-seq peaks-- containing the motif
Percent.of.Background.Sequences.with.Motif	The percent of regions in the background--here all ATAC-seq peaks--containing the motif
