# Supplementary material for: Altered transcriptional and chromatin responses to rhinovirus in bronchial epithelial cells from adults with asthma
Source: Commun Biol. 2020 Nov 13;3:678. doi: 10.1038/s42003-020-01411-4 (PMC7666152; doi:10.1038/s42003-020-01411-4)
Supplement: Supplementary file 3 — Description of Additional Supplementary Files [file 42003_2020_1411_MOESM3_ESM.pdf]

Description of Additional Supplementary Files

File name: Supplementary Data 1

Description: Clinical Demographics for Bronchial Epithelial Cells

File name: Supplementary Data 2

Description: RV-Response in Cases and Controls for All Expressed Genes

File name: Supplementary Data 3

Description: Assessment of GO Terms Enriched in RV-Response in Cases or Controls and Asthma in Vehicle- or RV-Treated Samples

File name: Supplementary Data 4

Description: RV-Response to Areas of Open Chromatin in Cases and Controls

File name: Supplementary Data 5

Description: Enrichment of Transcription Factor Binding Motifs in Areas of Open Chromatin in Cases and Controls in both Increased Accessibility and Decreased Accessibility Sites

File name: Supplementary Data 6

Description: All Hi-C Loops with Quality Scores above Five

File name: Supplementary Data 7

Description: RV-Responsive Areas of Open Chromatin and Genes Expression in Controls, Pairs Defined by pcHi-C

File name: Supplementary Data 8

Description: RV-Response in Cases and Controls Across Asthma Genes Defined by GWAS (Pividori, 2019)
